# Investigation of Histone Lysine-Specific Demethylase 5D (KDM5D) Isoform Expression in Prostate Cancer Cell Lines: a System Approach

**DOI:** 10.7508/ibj.2016.02.007

**Published:** 2016-04

**Authors:** Zohreh Jangravi, Mohammad Najafi, Mohammd Shabani

**Affiliations:** 1 Dept. of Biochemistry, Iran University of Medical Sciences, Tehran, Iran;; 2 Dept. of Molecular Systems Biology, Cell Science Research Center, Royan Institute for Stem Cell Biology and Technology, ACECR, Tehran, Iran;; 3 Dept. of Biochemistry, Razi Drug Research Center, Iran University of Medical Sciences, Tehran, Iran

**Keywords:** KDM5D, Prostate cancer, Regulatory networks

## Abstract

**Background:**

It is now well-demonstrated that histone demethylases play an important role in developmental controls, cell-fate decisions, and a variety of diseases such as cancer. Lysine-specific demethylase 5D (*KDM5D*) is a male-specific histone demethylase that specifically demethylates di- and tri-methyl H3K4 at the start site of active gene. In this light, the aim of this study was to investigate isoform/transcript-specific expression profiles of *KDM5D *in three prostate cancer cell lines, Du-145, LNCaP, and PC3.

**Methods:**

Real-time PCR analysis was performed to determine the expression levels of different KDM5D transcripts in the prostate cell lines. A gene regulatory network was established to analyze the gene expression profile.

**Results:**

Significantly different expression levels of both isoforms were found among the three cell lines. Interestingly, isoform I was expressed in three cell lines while isoform III did only in DU-145. The expression levels of both isoforms were higher in DU-145 when compared to other cell lines (*P*<0.0001). The observed expression profile was determined by using regulatory network analyses.

**Conclusion:**

The present study, for the first time, not only showed the expression profiles of KDM5D isoforms in prostate cancer cell lines but also evaluated the effects of the gene regulatory network on the expression profile of this gene.

## INTRODUCTION

Lysine-specific demethylase 5D (KDM5D), so-called JARID1D, SMCY, and H-Y antigen, is a histone demethylase located on the Y chromosome in humans and most mammals^[^^1^^]^. Histone methylation and demethylation have been demonstrated to play an important role in gene regulation and biological events during the develop-ment of diseases, including neurological disorders and a variety of cancer types^[^^2^^]^. The emergence of histone lysine demethylases, as therapeutic targets, provides an opportunity to ensure the continuing fight against cancer^[^^3^^]^.

Similar to other KDM5 family members, KDM5D is able to demethylate di- and tri-methyl H3K4, suggesting that it may repress the expression of certain genes at the transcription level^[^^4^^]^. A deletion analysis of Y chromosome-specific genes in human prostate cancer revealed that the *KDM5D* gene is deleted in 52% of cases^[^^5^^]^, demonstrating its involvement in cancer pathogenesis. Prostate cancer, as the sixth leading cause of death in the world, can spread to certain areas of the body, including bones and lymph nodes^[^^6^^]^.

Amino acid sequencing has revealed that KDM5D is homologous to KDM5C (XE169, JARID1C, SMCX) with 86% identity and 91% similarity^[^^7^^]^. On the one hand, the same evolutionary origin implicates a functional equivalence of SMCY/SMCX^[^^1^^,^^8^^]^.

**Fig. 1:**
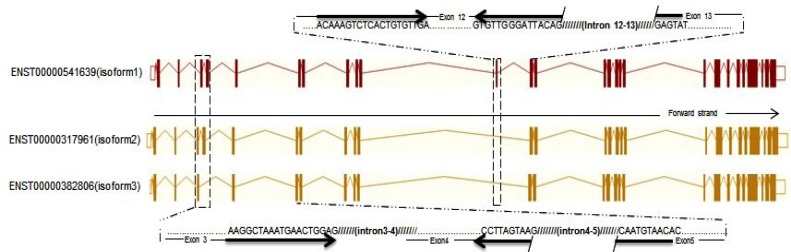
Three splice variants of KDM5D based on Ensembl database search. All isoforms have a common transcriptional start site but different numbers of exons. The difference in the number of exons has been marked with dashed box. The site of primers are shown in the Figure. Dashed lines indicate the exon site. Sequences of primers are highlighted with arrow. Slashed (///) lines show the intron position.

Figure 1 shows multiple transcript variants encoding three different isoforms. KDM5D isoform transcripts are found to be predominantly expressed in all male tissues. Most tissues, except spleen, exhibit differential expression of KDM5D transcript variants^[^^7^^]^. Alternative splicing of mRNA precursors allows many gene products with different functions to be produced from a single-coding sequence. In fact, this is one of the most significant aspects by which the genome acquires the ability to prime complex functional responses to different growth factors and environmental stimuli^[^^9^^]^. Several different mechanisms can contribute to changes in the regulation of transcript expression. Altered expression profiles or point mutations in components of the splicing machinery lead to different splicing patterns. An alteration in alternative splicing programs can be attributed to different diseases such as cancer^[^^9^^,^^10^^]^.

The principal aim of this study was to investigate the KDM5D isoform-specific expression pattern in three human prostate cancer cell lines (DU-145, PC3, and LNCaP) with different behaviors in tumourgenicity and other properties (Table 1). Furthermore, a gene regulatory network was established to evaluate the expression changes of factors corresponding to KDM5D, based on the microarray data and transcription factor profiles. 

## MATERIALS AND METHODS

Prostate cancer cell lines, Du-145, LNCaP, and PC3, obtained from the National Cell Bank of Iran (Pasteur Institute of Iran, Tehran), and cultured in RPMI 1640 medium (Gibco RL, Grand Island, NY) supplemented with 10% fetal bovine serum, 100 U/ml penicillin, 1% glutamine, and 1% non-essential amino acids in a humidified atmosphere containing 5% CO_2_. All of the cells were seeded at 60% to 80% confluency.

**RNA extraction and real-time PCR analysis**

Total RNA was extracted using Trizol reagent (Invitrogen, Carlsbad, CA, USA). The integrity and quality of RNA samples were assessed by a Nanodrop (ND-1000) spectrophotometer. Reverse transcription was carried out using M-MuLV reverse transcriptase (Fermentas Inc., MD, USA) from 1 µg of total RNA, according to the manufacturer’s protocol. Quantitative real-time PCR was performed to determine the expression of the KDM5D isoform with the SYBR Green PCR master mix (Applied Biosystems, Foster City, CA, USA) using an Applied Biosystems 7900 instrument. Relative mRNA levels were calculated using the comparative CT method as described by the manufacturer (Applied Biosystems, Foster City, CA, USA) with beta-actin as an internal control for normalization. Primer sequences used for quantitative real-time PCR are listed in Table 2.

**Table 1:** Some properties of prostate cancer lines used in this study

**Cell line**	**Tumorgenicity**	**Y** **chromosome**	**Androgen sensitivity**
PC3	High	delete	insensitive
DU-145	moderate	intact	insensitive
LNCaP	low	intact	sensitive

**Table 2:** Primer sequences

**Gene**	**Ref Sequence no.**	**Forward primer (5' to3')**	**Reveres primer (5' to3')**
KDM5D-isoform1	NM_001146705	ACAAAGTCTCACTGTGTTGA	ATACTCCTGTAATCCCAACAC
KDM5D-Isoform3	NM_001146706	AAGGCTAAATGAACTGGAG	GTGTTACATTGCTTACTAAGG
B-Actin	NM_001101	TCCCTGGAGAAGAGCTACG	GTAGTTTCGTGGATGCCACA

The primers were designed by Primer 3 (v. 2.2.3) and checked by NCBI primer BLAST

**Construction of** **gene regulatory** **network**

The Transfac data related to KDM5D was primarily extracted and then merged with protein-protein interaction (PPI) networks from PPI databases using Cytoscape software (ver. 3.1.1). The data extracted directly from the GEO records, GSE33455 and GSE32474, were transformed to the network nodes. The network edges were created according to the combined scores obtained from PPI data. 

**Statistical analysis**

All experiments were performed in triplicates, and the results were expressed as mean ± standard deviation (SD). Statistical analyses were performed using one-way ANOVA, followed by Tukey’s post-test. *P*<0.05 was considered to be statistically significant.

## RESULTS

**Expression of KDM5D isoforms in prostate cancer cell lines**

**Fig. 2:**
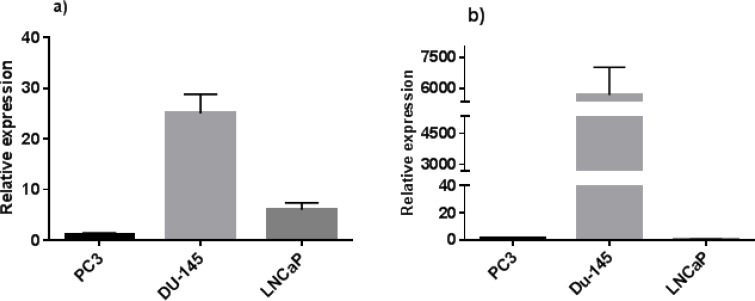
Relative expression of a) KDM5D isoform 1 and b) KDM5D isoform 3. Experiments were carried out in triplicate, and data were presented as mean ± SD. Both of isoforms are expressed in DU-145 higher than the other cell lines (*P*<000.1). The expression level of isoform1 in LNCAP is more than PC3 cell line (*P*<00.6

Real-time RT-PCR analysis was performed to determine the expression levels of different KDM5D transcripts in prostate cell lines, LNCaP, DU-145, and PC3 cells. Interestingly, the same expression pattern was found in both isoforms studied. As shown in Figure 2, the expression levels of these two isoforms in DU-145 cell line were found to be higher than the other cell lines (*P*<0.0001). KDM5D isoform I showed a minimum expression value in the prostate cancer cell lines PC3 and LNCaP, as illustrated in Fig. 2a. Furthermore, the expression level of isoform I in LNCaP was more than the cancer cell line PC3 (*P*<0.006). More importantly, KDM5D isoform III was expressed only in DU-145 but not in PC3 and LNCaP, as depicted in Fig. 2b.

**Network analysis**

**Fig. 3.**
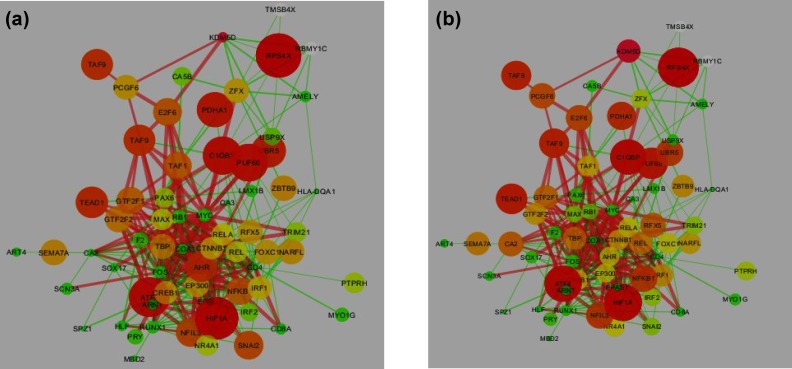
Gene regulatory network in a) PC3 and b) DU-145 cell lines. Green nodes and edges represent the lowest expression, red nodes and edges show the highest expression and the other colors indicate expression values between maximum and minimum. Size of nodes also indicate the level of gene expressions. Thickness of edges indicates the combined scores

To explain different expression patterns among different cell lines, we established a gene regulatory network corresponding to *KDM5D* gene expression in the cell lines. As shown in Figure 3, the networks share the same topology so that the size of nodes involved directly in KDM5D (including PCGF6, ZFX, RPS4X, USP9X, and E2F6) is not different between the cell lines. In addition, no significant dynamic difference was found between the networks. The edges between nodes representing individual molecular reactions were the same between the networks.

## DISCUSSION

In the present study, the specific primers for quantitative RT-PCR were developed to detect KDM5D isoforms-I and -III, and to investigate their expression pattern in the prostate cancer cell lines. Isoforms I and III encode proteins with a predicted molecular mass of 174 and 177 kDa, respectively, as a result of which Western-blot could not discriminate between the two isoforms.

It is well-known that isoform-level expression profiles provide better discrimination between cancer and non-cancer cells, as compared to the gene-level expression profiles. This is because of the fact that mammalian genes utilize different promoter elements and alternative splicing processes in various tissues, developmental stages, and cell lines^[^^11^^]^. However, the findings from the present study demonstrated that there is a significant difference in the expression level of both isoforms among different prostate cancer cell lines with different properties (Table 1). In addition, there is strong evidence that protein networks, including regulatory proteins, have the ability to determine the expression profile. To explain the different expression patterns between androgen-insensitive prostate cancer cell lines Du-145 and PC3, a *KDM5D* gene regulatory network was established based on the PPI data merged with Transfac data. Interestingly, no significant difference was found in network topology and dynamic output between the two cell lines. A network topology deﬁnes the connection between nodes while a dynamic gene network describes the change of gene expression at a given time^[^^12^^]^.

Lau and Zhang^[^^13^^]^ reported the expression of *KDM5D* and some Y chromosome genes in the cell line PC3. However, cytogenetic evidence demonstrated the lack of Y chromosome detection in the cell line. Therefore, they concluded that the aberrant expression of *KDM5D* and other Y chromosome genes in the cell line PC3 indicate that a part of the chromosome is in fact more likely to have been retained (or translocated to other chromosomes) in its genome. Nevertheless, we still cannot rule out the possibility that the RT-PCR products might be derived from the transcripts of the respective X alleles or homologues. 

A comparison between nodes and edges in the regulatory network in PC3 and DU-145 (Fig. 3) showed that there is no significant difference between regulatory networks governing the expression pattern of KDM5D in the prostate cancer cell lines studied. The results from this study demonstrated that the difference in the expression between cell lines is not related to that found in a regulatory factor. However, the aberrant expression of KDM5D derived from transcripts of the respective X alleles or homologues may be able to explain the difference in expression patterns of the cell lines.

The same expression pattern of two isoforms was found in Du-145 while the expression level of isoform I was demonstrated to be higher than that of isoform III in other cell lines. Both mutations in splicing proteins and point mutations in splice acceptor sites or donor sites led to different splicing patterns in a variety of transcripts^[^^10^^]^. 

Unlike the two other cell lines, LNCaP cells are androgen-sensitive human prostate adenocarcinoma cells^[^^14^^]^. The androgen receptor signaling axis plays a critical role in development, function, and homeostasis of the prostate^[^^15^^]^. The results from this study revealed that there is no correlation between the expression levels of the isoforms and androgen sensitivity or insensitivity. Although the three cell lines were considered to be different tumorgenicity, the present study demonstrated that there is no correlation between the isoform expression level and the level of tumorgenicity.

In conclusion, our results, for the first time, showed the isoform/transcript-specific expression of KDM5D in the prostate cancer cell lines. In addition, bioinformatics analyses identified the effect of gene regulatory network on the KDM5D expression profile in different cell lines.
